# Characterization of Schu S4 *aro* mutants as live attenuated tularemia vaccine candidates

**DOI:** 10.1080/21505594.2020.1746557

**Published:** 2020-04-02

**Authors:** Aimee L. Cunningham, Barbara J. Mann, Aiping Qin, Araceli E. Santiago, Christen Grassel, Michael Lipsky, Stefanie N. Vogel, Eileen M. Barry

**Affiliations:** a Center for Drug Evaluation and Research, FDA, Silver Spring, MD, USA; b Department of Medicine, Division of Infectious Diseases and International Heath, University of Virginia, Charlottesville, VA, USA; c Department of Pediatrics, University of Virginia, Charlottesville, VA, USA; d Center for Vaccine Development, University of Maryland Baltimore, Baltimore, MD, USA; e Department of Pathology, University of Maryland Baltimore, Baltimore, MD, USA; f Department of Microbiology and Immunology, University of Maryland Baltimore, Baltimore, MD, USA

**Keywords:** *Francisella tularensis*, tularemia, Schu S4, vaccine, *aroD*, dissemination, cytokines, pathology

## Abstract

There is a need for development of an effective vaccine against *Francisella tularensis*, as this potential bioweapon has a high mortality rate and low infectious dose when delivered via the aerosol route. Moreover, this Tier 1 agent has a history of weaponization. We engineered targeted mutations in the Type A strain *F. tularensis* subspecies *tularensis* Schu S4 in *aro* genes encoding critical enzymes in aromatic amino acid biosynthesis. *F. tularensis* Schu S4*ΔaroC*, Schu S4Δ*aroD*, and Schu S4Δ*aroC*Δ*aroD* mutant strains were attenuated for intracellular growth *in vitro* and for virulence *in vivo* and, conferred protection against pulmonary wild-type (WT) *F. tularensis* Schu S4 challenge in the C57BL/6 mouse model. *F. tularensis* Schu S4Δ*aroD* was identified as the most promising vaccine candidate, demonstrating protection against high-dose intranasal challenge; it protected against 1,000 CFU Schu S4, the highest level of protection tested to date. It also provided complete protection against challenge with 92 CFU of a *F. tularensis* subspecies *holarctica* strain (Type B). Mice responded to vaccination with Schu S4Δ*aroD* with systemic IgM and IgG2c, as well as the production of a functional T cell response as measured in the splenocyte-macrophage co-culture assay. This vaccine was further characterized for dissemination, histopathology, and cytokine/chemokine gene induction at defined time points following intranasal vaccination which confirmed its attenuation compared to WT Schu S4. Cytokine, chemokine, and antibody induction patterns compared to wild-type Schu S4 distinguish protective *vs*. pathogenic responses to *F. tularensis* and elucidate correlates of protection associated with vaccination against this agent.

## Introduction

*Francisella tularensis* is a Gram-negative coccobacillus and the etiological agent of the human disease tularemia which manifests clinically in multiple forms depending on the route of infection. *F. tularensis* is particularly lethal via the aerosol route, as inhalation of fewer than 10 CFU can initiate febrile illness [1,2]. Pulmonary tularemia is often misdiagnosed as it often initially presents with flu-like symptoms, and has a mortality rate of 30-60% if left untreated [3,4]. Proper antibiotic treatment reduces mortality to approximately 2% [5]. The low infectious dose and high mortality rate, coupled with its history of weaponization [5] demonstrates the need for a vaccine against this pathogen, which has been classified by the Centers for Disease Control as a Tier 1 Select Agent, a designation given to biological agents with the highest potential to be used as bioweapons that also pose significant risks to public health or agriculture.

Two subspecies of *Francisella* cause most diseases in humans. *F. tularensis* subspecies *tularensis* (Type A) causes the most severe form of disease, and subspecies *holarctica* (Type B) causes disease with reduced severity [6]. Previous attempts to develop a tularemia vaccine included the Live Vaccine Strain (LVS), an attenuated derivative of *F. tularensis* subspecies *holarctica* (Type B strain), which was tested extensively in humans [1,2]. Although LVS is not currently licensed for human use, it serves as proof of principle that a live attenuated vaccine can provide at least partial protection against subsequent challenge with the highly virulent wild-type (WT) *F. tularensis* subspecies *tularensis* (Type A). We proposed that generating rationally designed live attenuated vaccines in the subspecies *tularensis* background would result in an optimal tularemia vaccine that would provide protection against the most highly virulent subspecies that is responsible for the majority of human *F. tularensis* infections in North America [7,8]. We previously reported that vaccine candidates derived from subsp. *tularensis* with deletions in a series of targeted genes, including *guaB* and *guaA* genes, did not confer protection in the mouse model [9]. In this study, we have targeted another critical metabolic pathway by engineering three novel live attenuated vaccine candidates, Schu S4Δ*aroC*, Schu S4Δ*aroD*, and Schu S4Δ*aroC*Δ*aroD*. These metabolic mutants of the human virulent subspecies *tularensis* strain Schu S4 lack the functional proteins encoded by FTT0876 c (*aroC*), FTT0471 (*aroD*), or both genes. The *aro* genes encode enzymes of the shikimate pathway and have been mutated to attenuate other Gram-negative bacteria [10,11], including *Salmonella* [12–15]. This pathway is responsible for synthesis of aromatic amino acids in plants and microbes; deletion of *aro* genes has been empirically determined to decrease virulence in other organisms [10,13]. *F. tularensis* has been documented to produce all of the enzymes involved in the shikimate pathway [16], suggesting that this pathway would be an appropriate target with which to attenuate this pathogen.

All three vaccine candidates tested in this study were attenuated for growth within murine macrophages *in vitro*, and *in vivo* in C57BL/6 mice. Schu S4Δ*aroD* demonstrated the most promise, since administration of a prime-boost vaccine regimen to C57BL/6 mice provided protection against a pulmonary WT Schu S4 challenge of up to 1,000 CFU, one of the highest levels of protection documented to date in the mouse model. Additionally, Schu S4Δ*aroD* was protective against heterologous challenge with *F. tularensis* subspecies *holarctica* WT strain KY99-NR647. This vaccine strain was evaluated for further safety characteristics and host responses in vaccinated mice. This study identifies a promising, live attenuated vaccine candidate that is protective against high-dose respiratory challenge in mice and which may be useful in elucidating vaccine-associated correlates of protection against tularemia in the mouse model.

## Materials and methods

Bacteria. Schu S4 *aro* mutants were engineered by allelic exchange as previously described [9,17], using primers and plasmids described in Supplemental Tables 1 & 2. All primers are based on Genbank #AJ749949.2. Briefly, the suicide plasmid pFT893 was used to clone regions flanking *aroC* or *aroD* resulting in plasmids pFT938 or pFT985, respectively. Plasmid pFT938 contains a fragment encompassing regions 1.5-kb upstream and 1.5-kb downstream of *aroD* with the entire *aroD* gene deleted. Plasmid pFT985 contains a fragment encompassing regions 1.57-kb upstream and 1.46-kb downstream of *aroC* with the entire *aroC* gene deleted. Following electroporation of Schu S4, screening for gene deletions identified colonies in which an unmarked deletion was present in the target gene(s). Plasmids for trans complementation were engineered by amplifying the *aroC* or *aroD* gene from Schu S4 and ligating the respective genes into pFNLTP1 (kindly provided by Dr. Thomas Zahrt, Medical College of Wisconsin) to produce ppFT966 and pFT997. Wild-type *F. tularensis subsp. tularensis* strain Schu S4 and *F. tularensis subsp. holarctica* strain KY99-NR647 were obtained from BEI resources and preserved in aliquots at −80°C. Bacteria for both vaccination and challenge were grown in tryptic soy broth containing cysteine, as previously described [18]. Serial dilution plating to quantify inocula was performed on either tryptic soy agar supplemented with cysteine or Mueller Hinton agar (Becton Dickinson Microbiology Systems, Sparks, MD) supplemented with 1% glucose, 2.5% ferric pyrophosphate (both obtained from Sigma Chemical Co., St. Louis MO), 1% IsoVitalex (Becton Dickinson, Cockeysville, MD), and 10% defibrinated sheep blood. Studies to confirm auxotrophy were performed using chemically defined media (CDM) [19] supplemented with tryptophan (0.4 mg/ml) and phenylalanine (0.4 mg/ml) where indicated. Confirmation of the genetic stability of the Schu S4Δ*aroD* strain was performed by genomic sequencing and by PCR to detect the *aroD* deletion in both the bacterial stock and bacteria isolated from an infected mouse.

Intramacrophage replication assay. Intracellular replication of *Francisella* was evaluated as described previously [20]. J774A.1 cells (ATCC TIB-67) were seeded at a density of 3 × 10^5^ cells/well, and were infected with Schu S4 WT or mutant strains at an MOI of 100 for 2 hr. Following incubation, macrophages were washed 3x with dPBS, then were incubated for 1 hr in DMEM containing 50 µg/mL gentamicin to kill extracellular bacteria. Cells were washed again and maintained in DMEM media containing 2 µg/mL gentamicin for the remainder of the assay. At defined time points post-gentamicin treatment, cells were washed with dPBS and lysed with 0.02% SDS-PBS solution, and serial dilution plating on MHA agar was used to quantify intracellular bacteria.

Immunization protocol and WT challenge. WT 6-8-week-old female C57BL/6 mice were purchased from Jackson Laboratories (Bar Harbor, ME) and housed in a specific pathogen-free facility in the ABSL3 suite at the Univ. of Virginia. All animal experiments were carried out following the recommendations of the NIH Guide for the Care and Use of Laboratory Animals and were approved by the University of Virginia Institutional Care and Use Committee under protocol #3512. All efforts were made to minimize the suffering of the mice, with animals being euthanized upon reaching the first signs of terminal morbidity (clinical scoring rubric described in following section).

Groups of C57BL/6 mice (n = 3–7 mice per group) were anesthetized with ketamine-HCl-xylazine prior to intranasal (i.n.) inoculation with the indicated doses of Schu S4Δ*aroC*, Schu S4Δ*aroD*, Schu S4Δ*aroC*Δ*aroD*, wild-type (WT) Schu S4, or PBS. Mice were inoculated using a frozen stock that had been diluted to concentration that would provide the desired CFUs in 20 µl. The diluted stock was also plated in triplicate to determine the actual challenge dose. In Figure 4 mice that were determined to have received a Schu S4 dose of 1.5 CFU, were supposed to have received a dose of 20 CFU in 20 µl, but plating the inoculum revealed an actual average dose of 1.5 CFU. Booster doses were administered to some groups at the indicated time points. Where indicated, animals were subsequently challenged intranasally at 8 weeks post-priming with either WT Schu S4 (subspecies *tularensis*), or *F. tularensis* susp. *holarctica* strain KY99-NR647. All inoculation, vaccination, and challenge doses are indicated in Tables 1–3. After vaccination or challenge, mice were monitored daily for morbidity and mortality and euthanized at the first signs of terminal morbidity, typically a score of “2.” Clinical scores were assigned as follows: 0 = healthy mouse with normal behavior (exploring cage, feeding, alert, well groomed), 1 = mild illness (decreased movement, reduced grooming), 2 = moderate illness (decreased motion, eye closure, hunched posture), 3 = severe illness (motionless, eye closure, increased respirations, ruffled fur), 4 = moribund (no motion in response to external stimuli + criteria for (3)) or dead.

**Table 1.** Attenuated virulence of Schu S4 *aro* mutant strains.

Schu S4 Derivative Strain	Inoculation Dose (CFU) (i.n. route)	Survival Ratio	Time to Death (days)
Schu S4	2x10^3^	0/4	5,5,5,5
Schu S4Δ*aro*C	1x10^3^	4/4	>28,>28,>28,>28
Schu S4Δ*aro*C	1x10^4^	4/4	>28,>28,>28,>28
Schu S4Δ*aro*C	1x10^5^	4/4	>28,>28,>28,>28
Schu S4Δ*aro*C	1x10^6^	3/3	>28,>28,>28,>28
Schu S4Δ*aro*C	1x10^7^	3/3	>28,>28,>28
Schu S4Δ*aro*C	1x10^8^	3/3	>28,>28,>28
Schu S4Δ*aro*D	5x10^4^	4/4	>28,>28,>28,>28
Schu S4Δ*aro*D	1x10^5^	4/4	>28,>28,>28,>28
Schu S4Δ*aro*D	5x10^5^	0/4	10,10,10,10
Schu S4Δ*aro*D	1x10^6^	0/4	9,9,9,9
Schu S4 Δ*aro*C Δ*aro*D	1x10^8^	4/4	>28,>28,>28,>28
Schu S4 Δ*aro*C Δ*aro*D	3x10^8^	12/12	All >28

**Table 2.** Protection against WT SchuS4 challenge following i.n. vaccination with Schu S4 *aro* mutant vaccine strains.

Immunizing Strain	Prime Dose (CFU)	Booster Dose (CFU)	Challenge Dose (CFU)	Survival Ratio	Time to Death* (days)
PBS			100	0/4	5, 5, 5, 5
Schu S4∆*aro*C	1 x 10^6^		133	0/3	5, 5, 5
	1 x 10^7^		133	0/3	5, 5, 5
	1 x 10^8^		133	0/3	6, 6, 7
	1 x 10^8^	1 x 10^9^	90	2/3	7
	1 x 10^9^	1 x 10^9^	90	2/4	6, 7
Schu S4∆*aroD*	5 x 10^1^		100	4/5	7
	5 x 10^2^		100	4/5	10
	5 x 10^3^		100	5/5	
	1 x 10^4^		100	5/5	
	1 x 10^5^		100	5/5	
	5 x 10^1^	1 x 10^5^	250	5/5	
	5 x 10^2^	1 x 10^5^	250	5/5	
	5 x 10^3^	1 x 10^5^	250	5/5	
	5 x 10^1^	1 x 10^5^	500	4/5	9
	5 x 10^2^	1 x 10^5^	500	5/5	
	5 x 10^3^	1 x 10^5^	500	5/5	
	5 x 10^1^	1 x 10^5^	1000	2/5	7,7,7
	5 x 10^2^	1 x 10^5^	1000	1/5	5,7,9,12
	5 x 10^3^	1 x 10^5^	1000	1/5	6,6,6,9
	1 x 10^4^	1 x 10^8^	500	4/4	
	5 x 10^3^	1 x 10^8^	1000	5/5	
	1 x 10^5^	1 x 10^8^	1000	4/4	
Schu S4∆*aroD*	PBS		150	0/3	5,5,5
	1 x 10^3^	1 x 10^5^	150	5/5	
	1 x 10^3^	1 x 10^5^	750	5/5	
Schu S4Δ*aroC*Δ*aroD*	1x10^8^		28	1/4	6, 6, 6

**Table 3.** Vaccination with Schu S4Δ*aro*D confers protection against lethal challenge with homologous and heterologous virulent *F. tularensis* strains.

Group #	Priming Dose (CFU)	Booster Dose (CFU)	Date of Boost (weeks)	Challenge Dose (CFU)	Challenge Strain	Survival
1	-	-	n/a	42	Schu S4	0/3
2	451	-	n/a	42	Schu S4	7/7
3	451	5x10^5^	5	42	Schu S4	7/7
4	451	5x10^5^	5	420	Schu S4	7/7
5	451	5x10^6^	5	420	Schu S4	6/7
6	451	5x10^5^	5	92	KY99-NR647	7/7
7	-	-	n/a	92	KY99-NR647	0/3

Bacterial Dissemination and Cytokine Studies. Mice (n = 18 per group) were inoculated i.n. with 5.6 × 10^3^ CFU of Schu S4Δ*aroD*, or 1.5 CFU WT Schu S4, or received PBS as a mock vaccination. At 1, 3, 7, 14, and 21 days post-inoculation, mice (n = 3 per group/time point) were euthanized and the lungs, and livers harvested for determination of bacterial burdens, cytokine gene transcriptional analyses, and histopathology. Tissues were homogenized using disposable tissue grinders (Fisherbrand) and serial dilution plating was performed to quantify bacterial burdens in individual animal organs as described above for determining the inoculation dose.

Sections of livers were prepared for histological evaluation. Sections of formalin preserved organs embedded in paraffin were stained with hematoxylin and eosin stain (H&E) and scored in blinded fashion for histopathology [21].

Pieces of lungs were removed aseptically and homogenized in Trizol reagent (Ambion, Carlsbad, CA) using a tissue homogenizer (Omni International, Kennesaw, GA). RNA was extracted following manufacturer’s protocols, DNase-treated using a Turbo DNase kit (Ambion), re-precipitated and stored at −80°C. cDNA was prepared from 1 µg of RNA using the cDNA Synthesis kit (Quanta Biosciences, Gaithersburg, MD) per the manufacturer’s instructions. Levels of murine mRNA for specific cytokine and chemokine genes were quantified using quantitative Real-time reverse transcription PCR (qRT-PCR) as previously described [22], and are reported as relative fold change over background levels detected in mock-challenged animals (*i.e*. those that received PBS). Genes that were not induced in PBS-inoculated animals were used as background levels. qRT-PCR was performed in triplicate using an ABI Prism 7900 Sequence Detection System (Applied Biosystems, Foster City, CA) using primer sets and reagents previously described [22,23]. Hypoxanthine guanine phsphoribosyl transferase (HPRT) was used as a housekeeping gene for normalization of all samples.

Humoral Responses to Vaccination. Blood was collected on day 21 post-inoculation from a subset of vaccinated mice to obtain sera for antibody titration by ELISA. Briefly, sterile Schu S4 antigen (NR-15753, obtained from BEI Resources) was diluted in PBS (10^7^ CFU/mL, 100 µL/well) and used to coat wells of 96-well plates overnight at 4°C. Plates were washed 3x with PBS + 0.05% Tween-20 and blocked with PBS + 10% nonfat dry milk for 1 hr. Sera was serially diluted in blocking buffer starting at 1:100 dilution, incubated for 2 hr at room temperature, washed again, and incubated for 1 hr at room temperature with secondary antibodies (goat anti-mouse Ig, IgM, IgG1, IgG2 c, or IgA, diluted 1:250 in blocking buffer [Southern Biotech, Birmingham, AL]). Plates were washed again, developed with tetramethylbenzidine (TMB) substrate (KPL, Gaithersburg, MD), and stopped using 1 M phosphoric acid. Plates were read on a Versamax tunable microplate reader (Molecular Devices, Sunnyvale, CA) and endpoint titers were calculated for each individual animal.

Co-culture assay. Co-culture assays were performed essentially as described [24] but with WT Schu S4 instead of LVS. Bone marrow-derived macrophages from C57BL/6 J mice were seeded at 2 × 10^6^ cells/well in 24-well plates and infected with Schu S4 at an MOI of 0.1. Following a 2 hr incubation, cells were washed with gentamicin and resuspended in media without antibiotic. Single-cell suspensions of splenocytes (5 x10^6^/well) derived from mice immunized with Schu S4Δ*aroD* or PBS were added to infected macrophages. Splenocytes were derived from three mice per immunization group and splenocytes from each individual mouse were added to triplicate wells of infected macrophages. Following 24- or 48 h of co-culture, cells were washed, lysed, and intracellular bacteria quantified by serial dilution.

Statistics. Statistical analysis was performed using GraphPad Prism 6 software, and figures illustrate the mean ± standard error of the mean. Two-way ANOVAs with multiple comparisons with Tukeys post hoc test between log-transformed bacterial CFU counts were used to assess statistical differences in intramacrophage replication.

## Results

### Characterization of Schu S4 *aro* mutants

Unmarked deletions in the FTT0876 c (*aroC*) and FTT0471 (*aroD*) genes were created in *F. tularensis* strain Schu S4 using a two-step allelic exchange strategy as previously described [9]. PCR (Suppl. Figure 1) and sequencing were used to confirm the absence of the WT genes in both single mutants. To generate the double mutant *Ft* Schu S4Δ*aroC*Δ*aroD*, the Schu S4Δ*aroC* mutant was selected for introduction of the second mutation in the *aroD* locus. A selected Schu S4Δ*aroC*Δ*aroD* mutant strain was analyzed by PCR (Suppl. Figure 1). Amplification of the intact *aroD* and *aroC* genes was observed in the WT strain while the deleted versions of *aroD* and *aroC* were observed in Schu S4Δ*aroC*Δ*aroD*.

The Schu S4 *aro* mutants grew well in rich media. The auxotrophy resulting from mutations in *aro* genes was confirmed by testing growth in chemically defined media (CDM). WT Schu S4 grew well in CDM, but the mutants grew in CDM only when supplemented with phenylalanine and tryptophan.

**Figure 1.**
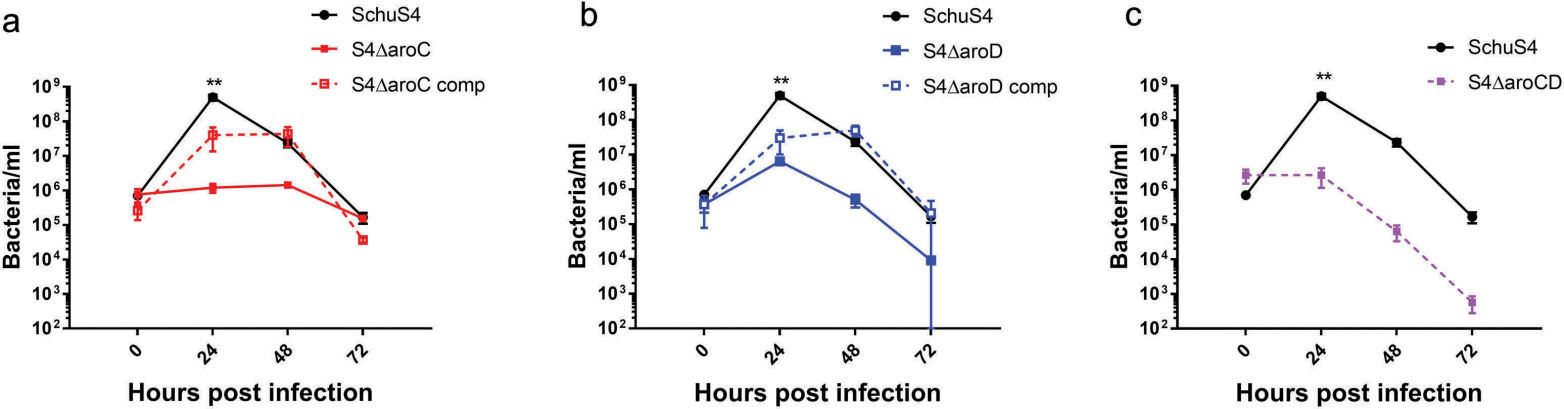
Schu S4 *aro* mutants are attenuated for growth in murine macrophages. Murine J774A.1 macrophages were infected with *F. tularensis* WT strain Schu S4 or Schu S4Δ*aroC* (panel a), Schu S4Δ*aroD* (panel b), or Schu S4 Δ*aroD*Δ*aroC* (panel c) for 2 hr. Cells were washed in gentamicin-containing media and incubated for the indicated time periods. Intracellular bacteria were enumerated at 0, 24, 48, and 72 hr post-infection. Data are presented as means ± standard errors of means with three biological replicates for each experiment. Data are derived from a single representative experiment (n = 2–3). **p < 0.001 (average CFU were analyzed by two-way ANOVA).

The ability to replicate intracellularly in macrophages is a critical pathogenic feature of *F. tularensis*. The three *aro* mutants were significantly attenuated for intracellular growth in J774A.1 murine macrophage-like cells (Figure 1). WT Schu S4 replicated to high intracellular numbers during the first 24 hrs post infection, exhibiting 9 doublings in this time period. Schu S4Δ*aroD* and S4Δ*aroC* were recovered at significantly lower numbers at the 24 hr time point and exhibited 4 and 1 doublings, respectively. The double mutant, Schu S4Δ*aroC*Δ*aroD*, failed to replicate within the macrophages, and remained at initial invasion levels at the 24 hr time point.

At 48 hr post-infection, WT Schu S4 numbers decreased by ~10-fold, due to increased cellular damage and cell death, but remained higher than the mutant strains. Trans-complementation of each respective homologous gene in the S4Δ*aroC* and S4Δ*aroD* strains restored growth in macrophages to near WT levels. The double Schu S4Δ*aroC*Δ*aroD* mutant was not complemented since the two genes could not be cloned and effectively expressed in a single plasmid.

*In vivo* attenuation of the mutant strains was assessed using the C57BL/6 J mouse model of i.n. infection. A series of escalating doses of each mutant strain was administered to groups of 3–4 mice, which were assessed for morbidity and mortality compared to WT-infected mice (Table 1). All three mutants were attenuated compared to WT Schu S4, which is lethal at a dose of 1–10 CFU [25]. In contrast, the LD_50_ for Schu S4Δ*aroC* and Schu S4Δ*aroCaroD* were >10^8^ CFU. The LD_50_ for Schu S4Δ*aroD* was between 1 × 10^5^ and 5 × 10^5^ CFU. Thus, Schu S4Δ*aroD* was significantly more attenuated than WT Schu S4 but less attenuated than the other two mutants.

### Schu S4Δ*aro*D protects against subsequent intranasal WT challenge

The attenuated virulence of these *aro* mutant strains made them candidates for consideration as vaccines and their ability to confer protection against lethal i.n. challenge with WT Schu S4 was tested (Table 2). Although Schu S4Δ*aroC* and Schu S4Δ*aroC*Δ*aroD* have high levels of attenuation, single-dose vaccination regimens with doses as high as 10^8^ CFU failed to protect mice against subsequent WT challenge. However, an i.n./i.n. prime-boost vaccination regimen of Schu S4Δ*aroC* provided partial protection (50-66%) against a 90 CFU WT challenge. In contrast, immunization with a single dose of 50 CFU Schu S4Δ*aroD* provided 80% protection against challenge with 100 CFU WT Schu S4. Increasing the single dose of Schu S4Δ*aroD* to 5,000 CFU conferred 100% protection against an identical WT challenge dose. Expanded immunization/challenge studies were performed with Schu S4Δ*aroD* to determine dosing parameters that could confer protection against high challenge inocula (250, 500, or 1000 CFU) of WT Schu S4. Adding a booster dose of 10^5^ CFU following the 50 CFU priming dose increased protective capacity of Schu S4Δ*aroD* and conferred full protection against challenge with 250 CFU WT Schu S4 and 80% and 40% protection against challenge doses of 500 and 1,000 CFU, respectively. Increased priming doses to 500 or 5,000 CFU followed by a 10^5^ CFU booster were completely protective against 250 or 500 CFU challenges. Increasing the priming dose to 10^3^ or 10^5^ allowed a boosting dose of 10^8^ CFU and conferred full protection against a challenge dose of 1,000 CFU (>100 LD_50_) (Table 2). Notably, these levels of protection against i.n. challenge with the highly human virulent Type A *Ft* in the mouse model have not previously been reported.

**Figure 2.**
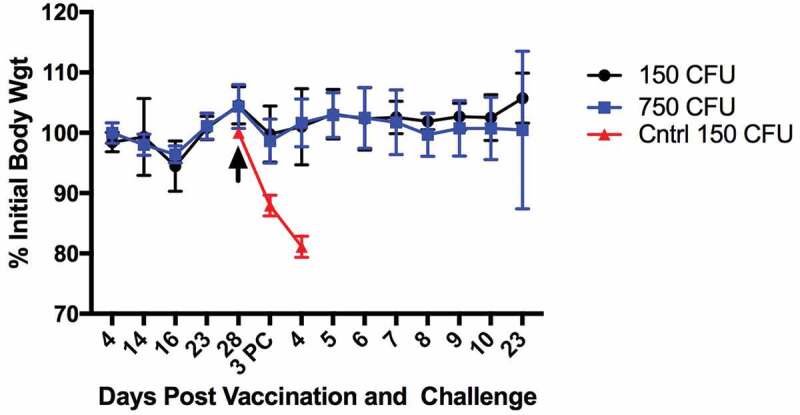
Mice i.n. immunized with Schu S4Δ*aroD* and challenged with WT do not exhibit weight loss. Mice were immunized and boosted by the i.n. route with 10^3^ and 10^5^ CFUs of Schu S4Δ*aroD*, respectively, then challenged (black arrow) i.n. with 150 (black circles) or 750 (blue squares) CFU WT Schu S4 on day 28 post-vaccination (n = 5 mice/group). Non-vaccinated mice were challenged with 150 CFU of WT Schu S4 on day 28 (red triangles). PC: post challenge.

A dose of 10^3^ CFU Schu S4Δ*aroD* was well within the range of both safe and immunogenic doses and was chosen for further evaluation. A second trial using immunizing and boosting doses of 10^3^ and 10^5^ CFUs, respectively, followed by an i.n. challenge of 150 or 750 CFUs on day 28 with WT Schu S4, conferred 100% protection (Table 2). Furthermore, in vaccinated mice, there were no significant changes in weight on days 4–28 after vaccination, nor during the 23 days after challenge (Figure 2). Vaccinated and challenged mice also had no visible changes in appearance of the coat or eyes, or in activity levels. In contrast, unvaccinated controls rapidly lost weight and succumbed to infection by day 4 post challenge. Using a Two-way ANOVA the weights of all mice from day to day were not significantly different, with the exception of days 16 and 28. Since all mice were equally affected on these days this was likely a weighing or scale error. Together, these results support the conclusion that immunization with Schu S4Δ*aroD* is generally well tolerated.

### Schu S4Δ*aro*D induces humoral and cellular immune responses

**Figure 3.**
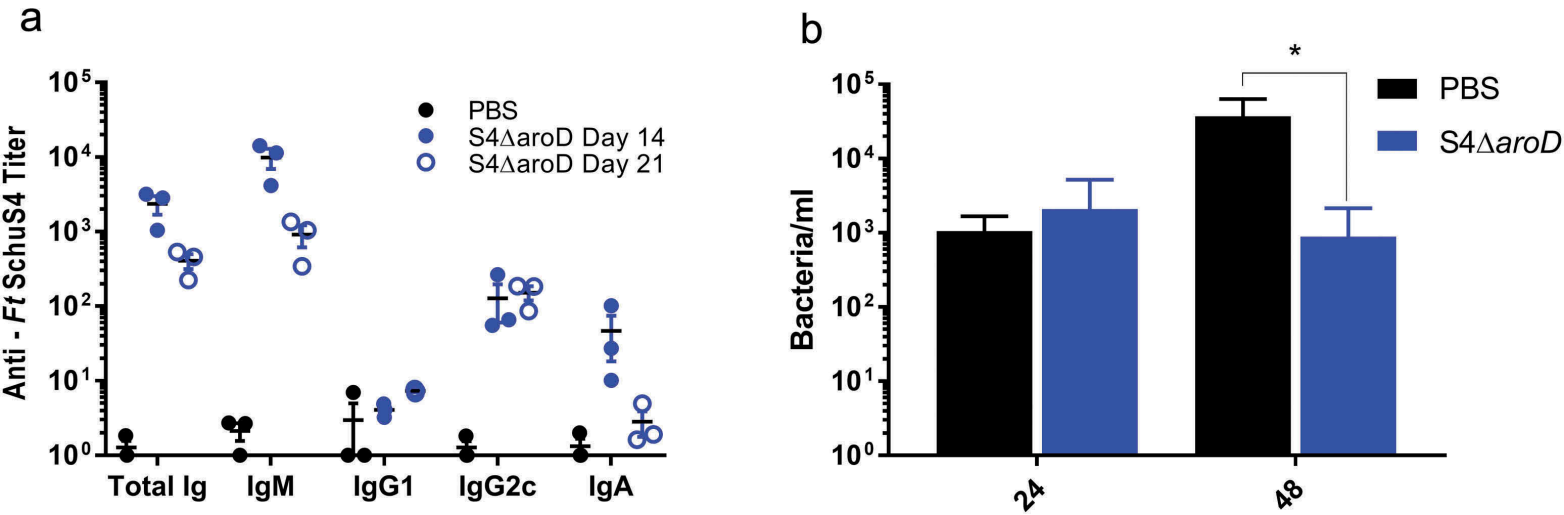
Immune responses to vaccination with Schu S4Δ*aroD*. Mice were immunized with Schu S4Δ*aroD* or PBS by the i.n. route. (a) Blood was collected on days 14 and 21 post-inoculation and serum antibody titers against an inactivated whole cell lysate of SchuS4 were determined by ELISA for individual mice. (b) Splenocytes were isolated (day 21 post-vaccination) and tested for the ability to suppress intracellular replication of WT Schu S4 in murine macrophages following co-culture. Data are presented as means ± standard error of means from triplicate wells for each of three mice per treatment group. Data are derived from a single representative experiment (n = 2). *p < 0.0001 (average CFU were analyzed by two-way ANOVA).

Since Schu S4Δ*aroD* showed the most promise as a tularemia vaccine candidate, we chose to further characterize this strain in pre-clinical studies using the murine model. The humoral responses to vaccination with of Schu S4Δ*aroD* were determined by quantifying day 14 and day 21 serum titers to a Schu S4 lysate. All vaccinated animals mounted a robust antibody (total IgG and IgM) response to whole cell *F. tularensis* lysates by day 14 post-immunization (Figure 3a). Serum IgM and IgA titers decreased between days 14 and 21, while IgG2 c remained elevated and IgG1 remained unchanged from baseline levels. Mice mounted a predominantly Th1-type immune response (IgG2 c > IgG1) systemically with little IgA in sera following immunization.

Work by De Pascalis and Elkins [24,26] established an *in vitro* co-culture system that measures functional T cell responses that correlate with vaccine protection. In this model, splenocytes from immunized mice are co-cultured with macrophages infected with *F. tularensis*. The ability of the immune splenocytes to control intra-macrophage replication correlates with the protective capacity of the vaccine. Splenocytes harvested from mice immunized with Schu S4Δ*aroD* on day 21 post-vaccination significantly inhibited WT Schu S4 intracellular replication in mouse macrophages compared to splenocytes from PBS-immunized mice (Figure 3b).

### Attenuated growth of Schu S4Δ*aro*D in mice following i.n. inoculation

**Figure 4.**
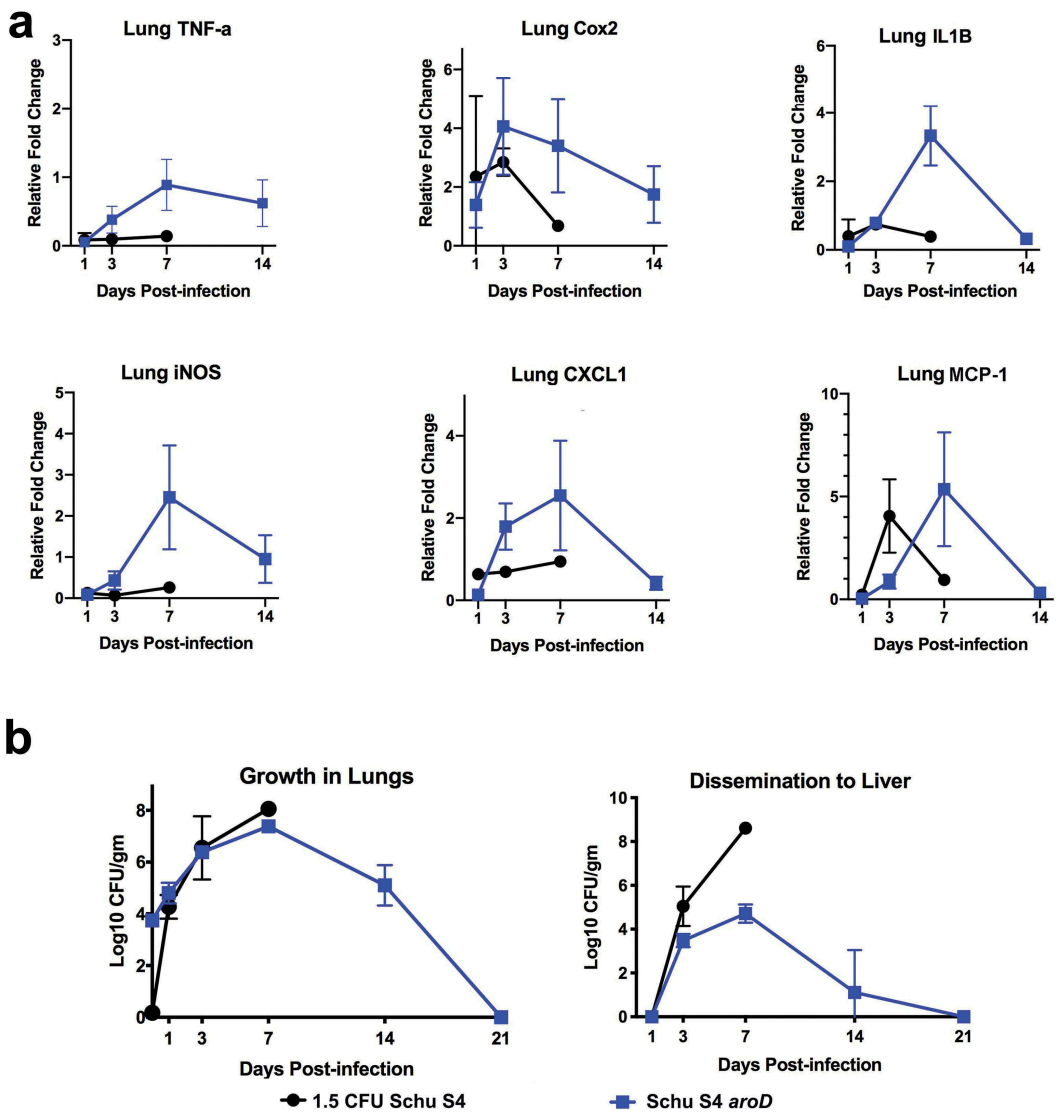
Modulation in cytokine gene induction levels in lungs following i.n. inoculation with Schu S4Δ*aroD* or WT Schu S4. C57BL/6 mice (n = 3 per group at each time point) were inoculated i.n. with 5.6 × 10^3^ CFU Schu S4Δ*aroD* or 1.5 CFU WT Schu S4 and euthanized on days 1, 3, 7, 14 post-infection. Tissue sections were preserved in Trizol reagent and processed to extract RNA, which was assayed by RT-PCR for murine TNF-α, COX-2, IL-1β, CXCL-1, iNOS, and MCP-1. All samples were assayed in triplicate and normalized to levels of the housekeeping gene HPRT. Relative fold change in gene expression as compared to PBS-infected animals is shown (Panel a). Tissue burdens of bacteria in the lungs and liver from the same mice are shown in Panel b.

To further explore the vaccine potential of Schu S4Δ*aroD*, the kinetics of dissemination to, and replication in lungs and liver, were assessed over a defined time course. Groups of mice were inoculated i.n. with either 1.5 CFU of WT Schu S4 or 5.6 × 10^3^ CFU of Schu S4Δ*aroD* (Figure 4b). Due to the low dose (1.5 CFU) of the WT inoculum, it appeared that some mice did not receive the dose as evidenced by no detectable bacterial counts in the organs of 1/3 and 2/3 mice assessed on days 1 and 7, respectively. Therefore, data from these mice were excluded from Figure 4b. WT Schu S4 replicated rapidly in the lungs of mice; at 24 hr post-inoculation, the numbers of bacteria increased by >4 logs from the initial inoculum of 1.5 CFU (Figure 4b). In contrast, after 24 hr, the number of Schu S4Δ*aroD* bacteria increased only by ~2 logs from the inoculating dose. WT-infected animals reached a high bacterial burden (10^7^ CFU/g tissue) in the lungs by day 3, and the one surviving infected animal on day 7 had a lung burden of 10^8^ CFU/g tissue. This mouse showed clinical signs of disease and was euthanized on day 7. In comparison, animals inoculated with 5,600 CFU of Schu S4Δ*aroD* had a peak lung burden of ~10^7^ CFU/g tissue on day 7, but showed no clinical signs of disease and completely cleared the bacteria by day 21. This peak burden of the Schu S4Δ*aroD* vaccine strain in the lungs is similar to that reported previously for LVS, a vaccine that is safe in humans, and highlights the sensitivity of the mouse model for *Francisella* [27,28].

Consistent with the high levels of bacteria in the lungs, WT Schu S4 also disseminated to the liver by day 3 post-inoculation where replication was uncontrolled and high burdens were measured (~3x10^5^ CFU/g tissue). The one mouse that survived to day 7 exhibited clinical symptoms and had an extremely high bacterial burden in the liver. In contrast, Schu S4Δ*aroD* disseminated to the liver by day 3 but did not reach the same high bacterial burden level as WT Schu S4 (~7x10^4^ CFU/g tissue) on day 7 and was completely cleared by day 21. None of the animals inoculated with Schu S4*aroD* showed clinical signs of disease.

**Figure 5.**
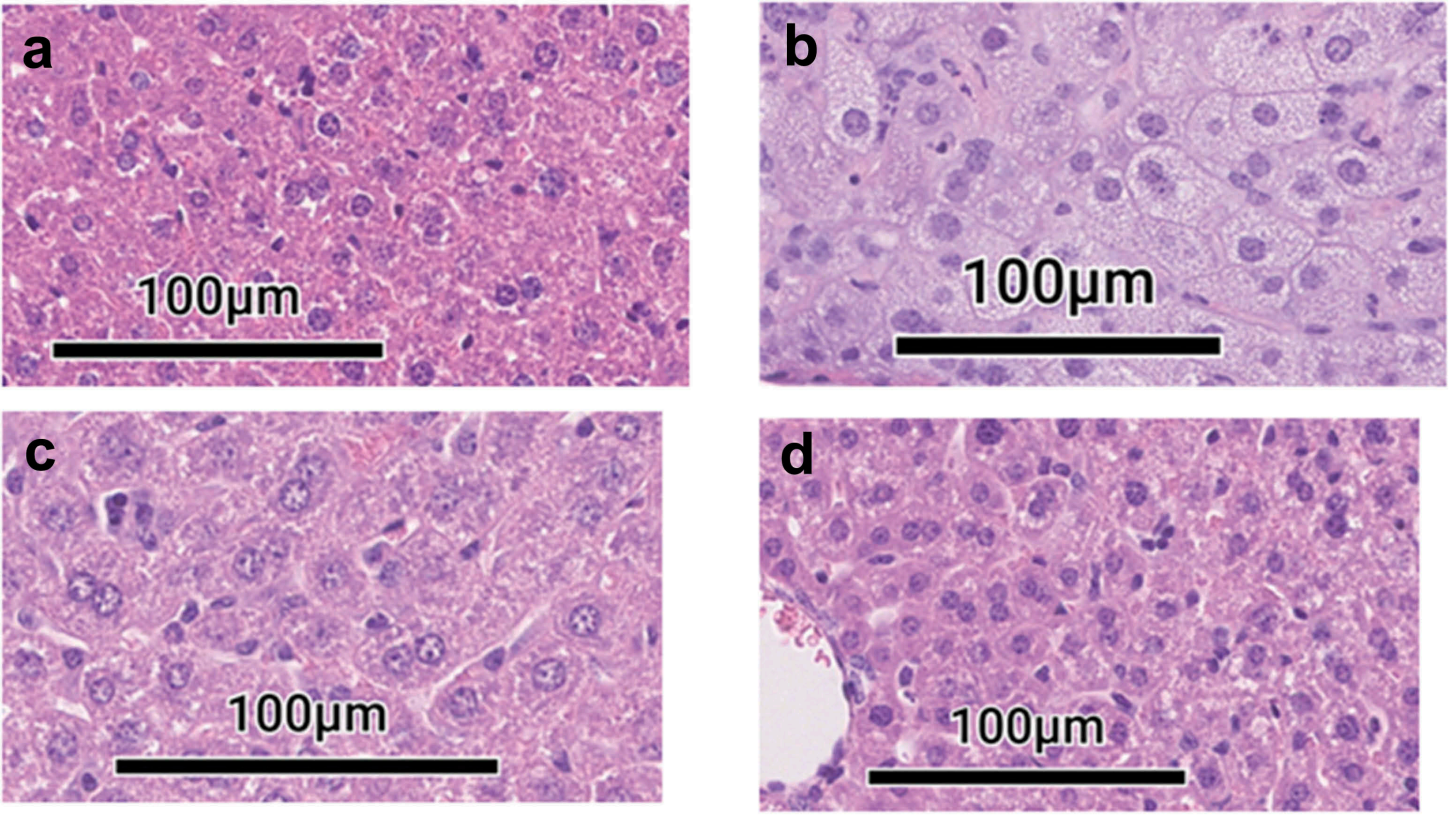
Pathology in livers of mice inoculated with S4Δ*aroD* vs WT Shu S4. C57BL/6 mice (n = 3 per group at each time point) were inoculated i.n. with 5.6 × 10^3^ CFU Schu S4Δ*aroD* or 1.5 CFU WT Schu S4 and euthanized on days 1, 3, 7, 14 post-infection. Liver sections were, stained with H&E. (a) Liver section taken from mock-infected mouse. (b)Liver section taken from WT Schu S4-infected mouse on day 7. (c, d) Liver sections taken from Schu S4Δ*aroD*-infected mouse on days 7 (c), and 21 (d) days post infection.

Histopathology analysis confirmed that liver pathology was consistent with burden data and clinical signs (Figure 5). Mice that received WT Schu S4 showed mild inflammation on days 1 and 3 post-infection, which progressed rapidly, with formation of larger foci of inflammation, autolysis of tissue and cellular necrosis on day 7 (Figure 5). Mice that received 5,600 CFU of Schu S4Δ*aroD* showed minimal liver pathology compared to WT Schu S4-induced damage and included smaller foci of inflammation on days 7 and 14 and with minimal pathology by day 21 when bacteria were undetectable. By day 21, livers of animals that received 5,600 CFU of Schu S4Δ*aroD* demonstrated resolution of infection, suggesting that the liver may be self-repairing as it cleared the vaccine strain at this time point [29].

Overall, this data demonstrates that Schu S4Δ*aroD* replicates within the murine host, but is highly attenuated for virulence *in vivo* compared to WT. Schu S4Δ*aroD*-infected animals attained bacterial burdens at the site of infection (lungs) equivalent to those animals receiving WT Schu S4, but did not succumb to infection with the attenuated strain and failed to exhibit clinical signs of disease. Schu S4Δ*aroD* was able to be effectively controlled in organs of dissemination (liver) where it did not reach levels of unchecked replication observed with WT Schu S4.

### Schu S4Δ*aro*D induces different cytokine/chemokine expression in the lungs compared to WT-infected mice

To explore further the ability of vaccinated mice to clear infection, we analyzed cytokine gene expression patterns following pulmonary inoculation with vaccine *versus* WT strains. The induction of genes encoding cytokines (including TNFα, Cox2, IL-1β, iNOS, CXCL1, and MCP-1) in the lungs was assessed in mice on days 1, 3, 7 and 14 using qRT-PCR (Figure 4a). WT Schu S4 infection induced minimal levels of cytokine transcription in the lungs, consistent with previous reports that this pathogen causes a delay in upregulation of inflammatory mediators during early time points following pulmonary infection, which is thought to facilitate replication and dissemination [30–32]. In contrast, cytokine gene transcription in mice inoculated with 5,600 CFU of Schu S4Δ*aroD* peaked on day 7, correlating with the time point at which maximum bacterial organ burdens were observed (Figure 4b). Thus, the ability to mount an inflammatory response in the lung may underlie the ability of vaccinated mice to control subsequent infection.

### Schu S4Δ*aro*D is protective against virulent homologous and heterologous challenge

Lastly, the breadth of protection induced by Schu S4Δ*aroD* against both human virulent subspecies of *F. tularensis*, as well as the sterilizing nature of protection was assessed. Mice were vaccinated by the i.n. route using a single dose or prime-boost schedule and challenged 8 weeks later with either Schu S4 or KY99-NR647, wild-type strains of subspecies *tularensis* and *holarctica*, respectively (Table 3). All unvaccinated animals succumbed to challenge with Schu S4 and KY99-NR647 by day 5–7 post challenge whereas in total 34/35 total animals vaccinated with any Schu S4Δ*aroD* regimen survived homologous (96% survival) or heterologous (100% survival) i.n. challenge. At 20 days post-challenge, no vaccinated animals had detectible challenge bacteria in their blood, and 4/34 mice had detectible bacteria in their livers (2/7 mice in Group 2 with 869 and 6829 CFU/g tissue, and 1/7 each from Groups 3 and 4, with 244 and 1724 CFU/g, respectively). None of the animals challenged with *F. tularenis* subsp. *holarctica* had detectable challenge organism in their livers. These data demonstrate that vaccination with Schu S4Δ*aroD* with an optimal prime-boost strategy was protective against both human virulent subspecies of *F. tularensis* and induces near-sterilizing immunity after challenge.

## Discussion

The production of auxotrophic strains as live attenuated bacterial vaccines is a classic strategy that has proven successful for multiple pathogens including *Salmonella* Typhi and *Shigella* [10–14,33]. In this report, we developed rationally defined mutations in the shikimate pathway of *F. tularensis*, leading to the creation of the Schu S4 mutants Δ*aroC*, Δ*aroD*, and Δ*aroC*Δ*aroD*. All three strains were highly attenuated in the mouse model, but Schu S4Δ*aroD* was determined to be the most successful vaccine candidate in a prime/boost regimen against both human virulent subspecies of *F. tularensis* in four separate challenge experiments. The finding that the Schu S4∆*aroC* and Schu S4∆*aroC*∆*aroD* strains were more highly attenuated, but less protective against WT challenge than Schu S4∆*aroD* is in keeping with the complexity of achieving optimal safety with maintenance of robust immune induction. Another metabolic mutant in *F. tularensis*, S4*purMCD* [34], was also shown to be highly attenuated for growth in macrophages as well as for virulence in mice. This strain was partially protective in the BALB/c mouse model against challenge with SchuS4.

Vaccination with Schu S4Δ*aroD* conferred protection against high dose WT Schu S4 challenge of 1,000 CFU. This level of protection has not been achieved previously for any tularemia vaccine candidate in the mouse model [35–42]. In fact, it is even possible that the protective capacity of this strain is higher, as we have not yet tested higher challenge doses. The ability of Schu S4∆*aroD* to protect against challenge with a lethal dose of a heterologous WT Type B strain further supports its promise as a broadly efficacious vaccine. Furthermore, we recently reported the protective capacity of a single scarification dose of Schu S4∆*aroD* in an outbred rabbit model where 83% of animals survived an aerosol challenge that was 11 times the LD_50_ [43]. Importantly, the rabbits remained healthy after vaccination with Schu S4∆*aroD*; there was only a transient rise in body temperature and a small eschar that resolved by day 14 [43,44]. These responses are similar to those observed with LVS vaccination. While only small numbers of rabbits were tested, evidence from this second animal model supports the potential of this vaccine for human use.

Correlates of protective immunity against tularemia have not been fully defined, but studies in the literature support the contribution of both antibody and cellular immune responses for protection against virulent *Francisella* [45]. Because vaccination with Schu S4Δ*aroD* induced both responses in mice, this vaccine will serve as a valuable tool in future studies to discriminate the specific protective mechanisms. It is noteworthy that vaccination with Schu S4 *aroD* induced a functional T cell response as determined in the splenocyte co-culture assay [24,26,46]. This assay perhaps is the best “correlate” of immunity thus far identified because it relies on antigen-specific T cells to produce IFN-γ leading to the activation of macrophages that control bacterial replication and survival by inducing anti-microbial and inflammatory genes. The induction and control of the expression of the iNOS gene may be one critical parameter which we observed in the lungs of Schu S4Δ*aroD*-vaccinated mice. In co-culture experiments, using splenocytes isolated from mice immunized a Schu S4 *clpB* mutant and bone marrow-derived macrophages (BMDMs) infected with Schu S4, Golovliov *et al*. showed that BMDMs isolated from iNOS knockout mice were unable to control the growth of Schu S4 [47]. iNOS is also required for mounting effective adaptive T cell responses; nitric oxide has been shown to influence T cell activation, differentiation, and memory [48]. Thus, nitric oxide may have both antimicrobial activity and promote the generation of a protective adaptive response through its effects on T cells.

One challenge of engineering live attenuated vaccines is achieving the optimal balance of attenuation and safety, while maintaining protective capacity. Bacterial growth and cytokine patterns in vaccinated animals are one way to assess this balance. On day 7, although the bacterial burden of Schu S4Δ*aroD* was similar to that for WT Schu S4 in the lungs, replication was beginning to be controlled in the liver. This was evident by the histopathology of liver, which showed minimal lesions in Schu S4Δ*aroD*-inoculated mice compared to a nearly obliterated organ in WT infected mice. After day 7, levels of Schu S4Δ*aroD* rapidly declined.

It is well recognized that one of the key virulence factors of *Francisella* is the ability to causes a delay in upregulation of inflammatory mediators during early time points following pulmonary infection is associated with enhanced replication and dissemination [30–32]. A potential key characteristic of vaccination with Schu S4Δ*aroD* is that cytokine expression levels increased until day 7, then declined. The levels of cytokine expression in the lungs was also directly proportional to the bacterial burdens in both the lungs and liver. This pattern of cytokine expression in the lungs is similar to that observed after vaccination with other attenuated, protective strains of *Francisella* such as LVS [49] and *clpB* [38,40]. These patterns of growth and cytokine responses may be required for an attenuated strain to elicit a high level of protective immunity and may explain why other attenuated strains such as the S4Δ*aroC*, S4Δ*aroCD*, and S4Δ*guaBA* [9] mutants were unable to protect against WT challenge. A high level of protective immunity for a tularemia vaccine would be important because, although natural infections with *F. tularensis*, either via a tick or contact with contaminated material, *are* likely to be low dose infections, a weaponized dose has the potential to be higher. Here, we have identified an attenuated Type A Schu S4-based vaccine strain that has the potential to protect against both natural and weaponized exposures. Future studies will include extended characterization of this candidate for safety, immunogenicity, and protective capacity using additional available models. While it is well beyond the scope of the current study, it would be important and informative to determine if vaccination with our most efficacious attenuated *F. tularensis* strain confers nonspecific resistance to infection with antigenically unrelated pathogens, as has been reported for another attenuated intracellular vaccine, BCG [50]. Like BCG, *F. tularensis* is highly macrophage-tropic. Thus, It is possible that our results reported herein reflect not only the induction of antigen-specific adaptive immune responses, but also, the activation of “trained immunity” elicited in innate immune cells (e.g. macrophages, dendritic cells) by epigenetic changes to histones that modify the subsequent transcriptional response to the same or unrelated pathogens.
