# Supplementary figures and images for: Characterization of Schu S4 aro mutants as live attenuated tularemia vaccine candidates
Source: Virulence. 2020 Apr 2;11(1):283–94. doi: 10.1080/21505594.2020.1746557 (PMC7161688; doi:10.1080/21505594.2020.1746557)

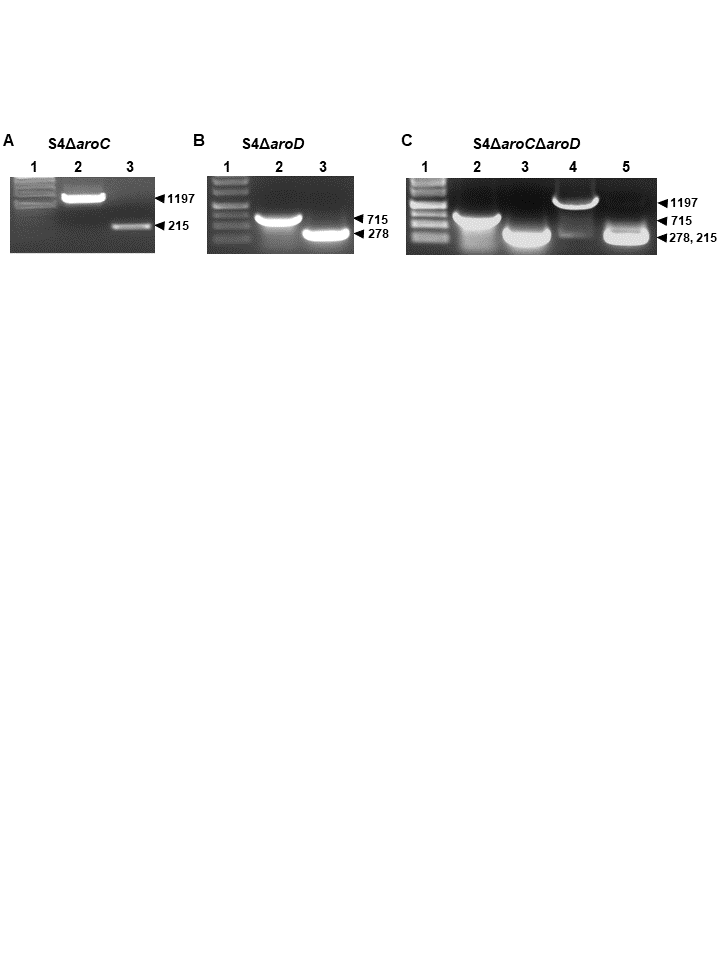

Supplement: Supplemental Material [file kvir-11-01-1746557-s001.png]
