# Supplementary material for: Characterization of Schu S4 aro mutants as live attenuated tularemia vaccine candidates
Source: Virulence. 2020 Apr 2;11(1):283–94. doi: 10.1080/21505594.2020.1746557 (PMC7161688; doi:10.1080/21505594.2020.1746557)
Supplement: Supplemental Material [file kvir-11-01-1746557-s002.docx]

**Supplemental Table 1.** Strains and plasmids used in this study

­­­­­­­­­­­­­­­­­**Strain/plasmid Relevant characteristics Source**

**WT strains**

*E. coli* DH5α *deoR, endA1, gyrA96, recA1, relA1, supE44* CVD

*F. tularensis* Schu S4 Wild type *F. tularensis* Type A strain ATCC/BEI

**Mutant strains**

Schu S4Δ*aroD*  Schu S4 mutant in FTT0471 locus This study

Schu S4Δ*aroC*  Schu S4 mutant in FTT0876c locus This study

Schu S4Δ*aroC*Δ*aroD*  Schu S4 mutant in FTT0471 and FTT0876c locus This study

**Precursor Suicide Plasmids**

pSacB pUC19 derivative containing sacB gene [[20](#_ENREF_20)]

pFT893 pSacBaphT derivative with P_guaB_-*sacB* fragment This study

pFT977 pFT893 derivative containing FTT0471 region This study

pFT938 pFT893 derivative containing FTT0876 region This study

**Suicide Plasmids**

pFT985 FTT0471 suicide plasmid This study

pFT954 FTT0876c suicide plasmid This study

***Trans*-complementation Plasmids**

pFNLTP1 Plasmid used to *trans*-complement mutants [[52](#_ENREF_52)]

pFT906 pFNLTP1 derivative with P_guaB_ promoter [[20](#_ENREF_20)]

pFT997 pFT906 derivative with full-length FTT0471 gene This study

pFT966 pFT906 derivative with full-length FTT0876c gene This study

**Supplemental Table 2.** Primers used in this study.

| **Name** | **Oligonucleotide Sequences (5’ → 3’)** | **Purpose (Schu S4 genomic region)** |
| --- | --- | --- |
| **ORF amplification including flanking regions** | | |
| P1400F | CGTAGCTAGGATCCAGGTGCTCTGACAAGATATTTGCTTTCTAGTAGTAAT | Amplification of the FTT0471 region (488002-491440) |
| P1401R | CGTAGCTAGGATCCCTTCACCCTTAGCAACTCTGATTTGCTCTTTGATAA |  |
| P1147F | CTGCGTGCTTGGATCCCACCTCCAAAATTTAGGTCATATCCTTTGTAATCATAC | Amplification of the FTT0876c region (883345-887283) |
| P1148R | CTGCGTGCTTGGATCCGTGTTCCATTGGTTACAACTATGGCATTGACCTTGC |  |
| **Deletion of the ORF by reverse PCR** | | |
| P1414F | GCTATGCCCCGGGGCAATAAATTACATAAATATGAAAGGAGAGTAG | Deletion of the FTT0471 ORF (489502-489939) |
| P1413R | GCTATGCCCCGGGGATTTGGTCCGTTAATAACTAAAACATCCAT |  |
| P1185F | ATGTCAGCCCCGGGGGTAACGATACTACTGTTATCACCAAAGGTAGACAT | Deletion of the FTT0876c ORF (884915-885819) |
| P1184R | ATGTCAGCCCCGGGACCAAAAGTATTTCCTGACATTATGCATAATAATAAGTAGCTTG |  |
| ***Trans*-complementation plasmids** | | |
| P1663F | TCATGCTACTAGTATGGATGTTTTAGTTATTAACGGACCAAATC | ΔFTT0471 *trans*-complementation (489501-489939) |
| P1664R | TACTAGCGTCGACCTACTCTCCTTTCATATTTATGTAATTTATTGC |  |
| P1358F | ATCCCGGGGCTAGCATGTCAGGAAATACTTTTGGTAAAATTTTTAC | ΔFTT0876 *trans*-complementation (884781-885839) |
| P1359R | ATCCCGGGGTCGACCTAATCCCTATAAGATCGCGTAATTAGGA |  |
| **Characterization of Schu S4 mutants by PCR** | | |
| P1661F | TAGCTTTAGATACAGGATGTGTGTTTGGA | Genotypic PCR for FTT0471; 715 bp (WT), 278 bp (Mutant) |
| P1662R | GCAACATTGCTAGCAACTGGTGCA |  |
| P1219F | GCTTCTAGTCTAATCAGTTATAG | Genotypic PCR for FTT0876c; 1197 bp (WT), 215 bp (Mutant) |
| P1221R | CCCAATCAATTAGTAAGCTTGAG |  |

**Supplemental Figure 1. Schu S4 *aro* mutants contain targeted unmarked deletions** . Genomic DNA was isolated from each mutant and WT strain and amplified by PCR with primers that flank the *aroC* (panel A), *aroD* (panel B) or both genes (panel C). Lanes 1, MW markers; lanes 2 and 4, WT Schu S4; lanes 3 and 5 *aro* mutant strains. Primers that flank *aroC* are P1661F and P1662R. Primers that flank *aroD* are P1219F and P1221R.
